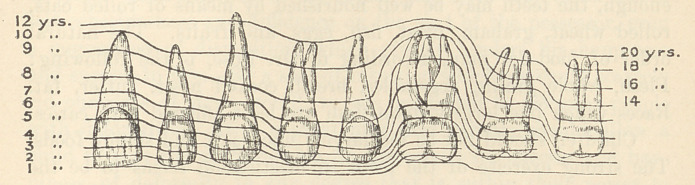# The Condition of the Teeth of Children in Public Schools

**Published:** 1901-07

**Authors:** G. E. Johnson

**Affiliations:** Andover, Mass.


### International Dental Journal.

Vol. XXII. July, 1901. No. 7.

### Original Communications.[1]

### THE CONDITION OF THE TEETH OF CHILDREN IN PUBLIC SCHOOLS.[2]

BY G. E. JOHNSON, ANDOVER, MASS.[3]

It is well known that with the advancement of civilization there has come an increasing tendency to physical degeneracy in many particulars. This is especially noticeable in regard to the jaws and teeth of the present generation. The teeth of Americans compared to those of contemporary savage tribes and half-civilized peoples, are seen at much disadvantage. Indeed, there has arisen a deep concern, not alone in this country, but in Europe[4] as well, over the conditions of the teeth of the rising generation. There is no small evidence that, should present conditions continue, a large class of people in this country would become toothless at a comparatively early period in life.

The regular practitioner has not a favorable opportunity, gen-

[1] The editor and publishers are not responsible for the views of authors of papers published in this department, nor for any claim to novelty, or otherwise, that may be made by them. No papers will be received for this department that have appeared in any other journal published in the country.

[2] Read before the American Academy of Dental Science, February 6, 1901.

[3] Superintendent of Public Schools, Andover.

[4] James Leon Williams: The Degeneration of the Human Teeth, New Review, vol. vii., 1892.

erally, for observing the condition of the teeth of all classes. To him, the actual condition of the teeth of the great mass of people is not known, except by inference, for the greater portion seldom or never consult a dentist. This is especially true in the case of children. A very great majority of even well-to-do parents do not employ a dentist for children with baby teeth, and doubtless most children would never see the inside of a dentist's office were it not for the purpose of having teeth extracted. Few dentists, therefore, are really in touch with a representative body of children. But there has been a deep interest growing among dental practitioners in this country and abroad in regard to the actual condition of the teeth of the rising generation. And the public school has offered the very best medium of all for gaining information on this matter. Here we find children of all ages, classes, and social conditions, and, we might almost say, of all nationalities, and it is not surprising that investigations of the nature of the one conducted at Andover should have been undertaken. It is rather surprising that far more extensive and earlier investigations have not been made.

In 1880 Dr. Samuel Sexton, Aural Surgeon to the New York Eye and Ear Infirmary, made a thorough examination of the teeth of eighty school children. Scarcely any of the children were free from dental irritation. "In thirty of the number," he says, "the teeth were in such an unhealthy state, from irregularities and decay, that wax impressions were taken of them; these have since been mounted up in plaster for study. The deplorable neglect of the teeth of these children was a surprise to me, although, from previous observations, I had expected to find them very bad indeed. It was notable that teachers having charge of these pupils never suspected that the teeth ever gave rise to any serious trouble, but it was ascertained by questioning the children themselves that in nearly every instance they had experienced pains in the teeth or ears, sometimes in both. The appearance of many of these children indicated that the general health had not escaped the consequences of imperfectly masticated food; that some of them also suffered from neuralgia about the head and face goes without saying."[1]

An investigation of this nature has been made in Dakota and

---

[1] Circular of Information of the Bureau of Education, No. 5, 1881, Washington.

Illinois. Six hundred and twenty-three children were examined, and thirty per cent. of all the teeth were found to be diseased.

Mr. Denison Pedley, in England, conducted an examination of the teeth of three thousand eight hundred school children from three to sixteen years of age. Seventy-five per cent. of the children had diseased teeth. About twelve per cent. of the teeth needed filling or extraction. While this was considered very bad, it is much better than the condition of American children thus far examined.

Unghavari, an Hungarian, in Scedegin, examined one thousand children between the ages of six and twelve, and found that 87.02 per cent. had diseased teeth. Twenty-two and one-half per cent. of the baby teeth were defective, 7.75 per cent. of the permanent teeth were defective.

In Hamburg 94.4 per cent. of three hundred and thirty-five orphan children had diseased teeth.

A very extensive investigation of the teeth of school children has been made by the association of dentists in the province of Schleswig-Holstein, Prussia. Nineteen thousand seven hundred and twenty-five children in nineteen cities were examined. Ninety-five per cent. of the children from six to fifteen years of age were afflicted with caries. Only two hundred and eighteen children of the nineteen thousand and over had ever been treated by a dentist. The mouth hygiene of these children was found to be very unsatisfactory, endangering the sound teeth and proving a source of infection.[1]

Dr. Karl Röse reports an investigation in the provinces of Baden and Thüringen of the condition of the teeth of school children. In the regions poor in lime, he finds, in Baden, 98.7 per cent. of the children afflicted, and 35.3 per cent. of the teeth diseased. In Thüringen ninety-eight per cent. of the children are afflicted, and 34.9 per cent. of the teeth are diseased. In regions rich in lime, he finds, in Baden, seventy-nine per cent.[2] of the children with diseased teeth, and 16.1 per cent. of the teeth affected; in Thüringen, 82.8 per cent. of the children with diseased teeth, and 16.7 per cent. of all the teeth diseased.

In Thüringen only twenty-seven children in six thousand three hundred and three had fillings. In Freiburg were found only fifty-

---

[1] Zeitschrift für Schulgesundheitspflege, No. 7, 1900.

[2] Ibid., No. 2, 1895.

three teeth out of twenty-eight thousand three hundred and forty-three saved by fillings. In the higher schools was found a better condition of the teeth in respect to care. In the Freiburg Gymnasium one diseased tooth in six was found to be filled.

Dr. C. Henie, school physician in Hamar, Norway, a town of about the size of Andover, examined six hundred and sixty school children from seven to fifteen years of age, and gives some very interesting tables which will be mentioned later in connection with my own.[1]

Four hundred and ninety-seven school children in Andover were examined,—two hundred and fifty-seven boys and two hundred and forty girls, from four years of age to eighteen. Ninety-six and nine-tenths per cent. of all the children were afflicted with caries. Only fifteen children, nine girls and six boys, had perfect teeth, and all but two of these were under nine years of age. Thirty-one and four-tenths per cent. of the teeth were diseased,—boys, thirty-two per cent.; girls, 30.8 per cent.; 41.7 per cent. of the temporary teeth, 26.5 per cent. of the permanent teeth.

TABLE OF DISEASED TEETH, BY AGES.

| Age, years. | Per cent. diseased. | Age, years. | Per cent. diseased. |
|---|---|---|---|
| 4 | 29 | 11 | 21 |
| 5 | 31 | 12 | 25 |
| 6 | 35 | 13 | 35 |
| 7 | 34 | 14 | 36 |
| 8 | 31 | 15 | 43 |
| 9 | 41 | 16 to 18 | 52 |
| 10 | 30 | | |

From the above will be noticed the general increase in the percentage of decayed teeth up to ten years of age, and then the quick drop during the next two years. Children between eight and nine have about one-half of their baby teeth still in the mouth, most of which are in bad condition. At ten years of age most of the baby teeth have given place to the new permanent teeth, and at eleven years of age children enjoy the greatest immunity from diseased teeth of any period in life after the fourth year. Alas that this

[1] Zeitschrift für Schulgesundheitspflege, No. 2, 1898.

more hopeful condition does not continue! At fifteen years of age they have lost all they had gained and more.

At about six years of age appears the first permanent molar. The fate of this first-born and natural heir of the strength and hardihood of the permanent teeth, as shown in the mouths of these children, is interesting. Evidently at its coming it is much needed,

TABLE COMPARING THE TEETH OF ANDOVER CHILDREN AND THOSE OF CHILDREN OF HAMAR, NORWAY.

| Age, years. | Per cent. diseased. Andover. | Per cent. diseased. Hamar. |
|---|---|---|
| 7 | 34 | 21.1 |
| 8 | 31 | 22.4 |
| 9 | 41 | 20 |
| 10 | 30 | 16 |
| 11 | 21 | 10.7 |
| 12 | 25 | 10.3 |
| 13 | 35 | 11 |
| 14 | 36 | 12.6 |
| 15 | 43 | 13 |

for most of the baby molars are now mere shells of bone, but a sad fate awaits it. Contaminated almost at once by its infected neighbors, we find this giant among the teeth diseased to the extent of one in every six, the very first year. Yet I find most eminent authority for the assertion that the sixth-year molar is naturally the strongest and most useful of all the teeth. Children between seven and eight have 40.4 per cent. of these teeth diseased; at eight, seventy per cent.; at twelve, seventy-eight per cent.; at fifteen, ninety per cent. Long before it is even suspected by many that these teeth are anything more than baby teeth, an error rudely dispelled when they come to the realization of the facts by having one pulled, very many are past help. Scott says of Zohauk, the Nubian slave, "The lord of speech has been stricken with silence betwixt the ivory walls of his palace." More fitly might the mouth of the American boy of nine or ten, with his shining new front teeth, be described as a sepulchre, white without, but within full of all uncleanness.

Dr. Mary E. Gallup, of Boston, for several years gathered statistics of the sixth-year molar in the mouths of native-born Americans. Of three thousand Americans over twenty-five years of age, only seven had all four sixth-year molars in the mouth.

TABLE SHOWING PROGRESS OF DECAY IN THE SIXTH-YEAR MOLAR.

| Age, years. | No. children. | No. molars. | No. diseased. | No. lost. | Per cent. diseased or lost. | |
|---|---|---|---|---|---|---|
| | | | | | Andover. | Hamar. |
| 5 to 6 | 36 | 30 | 4 | 0 | 13.3 | . . . |
| 6 to 7 | 32 | 92 | 14 | 0 | 15.2 | . . . |
| 7 to 8 | 27 | 105 | 47 | 0 | 40.4 | 18.5 |
| 8 to 9 | 34 | 136 | 90 | 2 | 70.2 | 40.9 |
| 9 to 10 | 51 | 204 | 143 | 7 | 73.5 | 50.3 |
| 10 to 11 | 50 | 200 | 131 | 17 | 74.0 | 52.8 |
| 11 to 12 | 56 | 224 | 132 | 19 | 67.4 | 49.3 |
| 12 to 13 | 60 | 240 | 152 | 37 | 78.7 | 58.3 |
| 13 to 14 | 51 | 204 | 131 | 36 | 81.8 | 55.5 |
| 14 to 15 | 26 | 104 | 81 | 16 | 93.2 | 57.5 |
| 15 to 16 | 15 | 60 | 41 | 13 | 90.0 | 60.3 |
| 16 to 18 | 15 | 60 | 43 | 13 | 90.0 | . . . |

Besides the decay of the teeth there were numerous abnormalities, some scarcely less serious than the disease of the teeth, either as regards appearance or health. More than one-fourth of the children examined—*i.e.*, twenty-six per cent.—had one or more of the following: Protruding upper or lower teeth, teeth pointing inward or outward, or jaws meeting at either front teeth or back teeth only, thereby interfering greatly with mastication of food. Two children were unable to bite the little finger when inserted between the front teeth. Some had teeth meeting end to end at the front of the mouth so that the molars were unable to touch those of the opposite jaw. Thirteen and one-tenth per cent. of these children had too long retained baby teeth, many of which were causing a crowding out of place of the coming second teeth. One day a little girl came to my office with a singular-appearing mouth, which, on my looking more closely, disclosed a double set of teeth across the entire front of the upper jaw, the baby set being allowed to remain unmolested in the way of the second set. All the baby teeth are normally displaced by the permanent teeth at about eleven years of age; yet we found many baby teeth in the mouths of children from twelve to fifteen years of age, and occasionally even to seventeen or eighteen years of age. Cases were not wanting of too early extracted baby teeth, and what is worse, of course, extracted by dentists.

Dr. W. H. Atkinson denounces this extraction of children's teeth as "murder," and claims that not five per cent. of children at sixteen years of age, in consequence, have fully and regularly developed jaws; while Dr. Edwin Collins, of the *London Dentist,* says that extraction of teeth should be scarcely less rare than amputation of limbs. And yet, not many years ago, in our enlightened Commonwealth it was not an unusual thing to see some travelling "dentist" extracting teeth, even of irresponsible children, in the public street by the dozens, "free of charge," and "without pain."

Three-fourths of the children examined in one building had unsightly stains upon the teeth. Of the one hundred and sixty-five children examined in this building, fifty-eight had prognathous upper jaw, eight prognathous lower jaw; twenty-eight had occlusion of molars and bicuspids only, ten of molars only; one hundred and thirty-six had green stains more [1] or less marked; forty-seven gave evidence of being mouth-breathers; twenty-two suggested the possibility of adenoids; eleven had abnormally high arches, three V-shaped arches.

The records written in these child-mouths also told us that, in the case of the great majority, it was only when afflicted with what Burns calls the "hell o' a' diseases," the toothache, that the dentist was ever employed, and then as one whose function it was to tear out these organs of digestion and rid the mouth of them, rather than to save them. The three hundred and twenty-six children over nine years old had lost one hundred and eighty-three permanent teeth,—more than one to every two children. After fourteen years of age there was an average of one permanent tooth lost to each child. One girl of fifteen had lost all her first permanent molars and one twelfth-year molar, and the other three had cavities.

It was very evident that many of these children had suffered much with the toothache. Twenty-two and one-half per cent. said they had suffered a great deal during the past year, and one-half of the remainder had suffered more or less.

While the percentage of cavities filled is much better than that found in Germany, yet it is amazingly small. Less than twenty per cent. of the cavities in the permanent teeth were filled. Among high-school pupils there was a better record, 50.3 per cent. of all cavities being filled.

Each mouth bore a testimony of its own in regard to the care

---

[1] Dental Cosmos, vol. iv.

taken of the teeth, but each child was also questioned on this point. Only thirteen per cent. of the children under six years of age brushed their teeth or had them brushed; eighty-seven per cent. rarely or never brushed their teeth. Of the whole number of children, only one in three made any pretence of regularly caring for the teeth. One might suspect that this low percentage was due to including in the calculation the very young children. But 62.9 per cent. of children over six years of age neglected to clean the teeth, and even twenty-three per cent. of the high-school pupils examined were guilty of like neglect.

So much for the condition of the teeth of school children in Andover. To recapitulate and make clear the general condition of the teeth, before passing to the next point, let us try to get a sort of composite picture of the average school child in Andover. He has twenty-four teeth; eight of them are diseased; sixteen of them are discolored with unsightly accumulation of food and deposits, or else he has some noticeable malformation, interfering with breathing or mastication, or disfiguring his appearance; three of the four first permanent molars are seriously affected, or else one is already lost and another decayed. He has either never put a tooth-brush to his teeth, and has had toothache more or less during the past year, or he is suffering excruciating pains, and has never seen the inside of a dentist's office. Furthermore, the chances, as will be shown later, are that he has suffered from malnutrition, that he is shorter and lighter than he should be, and that his school work has been impaired. And what is sadder, his condition is growing continually worse.

As I have said before, with the advancement of civilization there has been a corresponding degeneration of the human jaw and teeth. According to Dr. Röse, only 2.5 per cent. of Eskimos have defective teeth, three to ten per cent. of Indians, three to twenty per cent. of Malays, forty per cent. of Chinese, and eighty to ninety-six per cent. of Europeans; while ninety-seven per cent. of Andover school children are thus afflicted.

I am sure we do not wish to stop the advancement of civilization, but we should like to save the teeth. Just as it is true that man in his evolution from the lowest monkey has lost twelve teeth, so it may be that the race is yet to lose more teeth. Indeed, already four of the thirty-two teeth now considered the normal number for man give much evidence that they are about tired of appearing at all, and are ready to have their service to humanity called ended. Not to speak at length of the causes which combine against the teeth of man, we may briefly state, for the sake of what is to follow, the chief among them.

The changes in the physical structure of the body, incident to evolutionary progress; the lessening need of teeth as an initiatory organ of digestion; changes in the kind and composition of food; the general manner of living under modern social conditions, with its attending deteriorating effects; and, negatively, the fact that personal hygiene and care for the teeth have not advanced with sufficient rapidity to counteract these causes of physical degeneracy. For generations the brain has been encroaching upon the lower face and jaw. Only eight out of four hundred and two British soldiers had a width of jaw equal to the *average* of the Roman soldier, while the average American jaw is 0.37 of an inch narrower than that of the ancient Roman.[1] There is grave danger in these changes, the more because they are so imperceptibly gradual. But I do not believe that race development must be at the cost of the physical degeneracy of the individual. Modern education, modern science, medicine, and philanthropy are to triumph over these dangers and rescue the bodies of our children and of their children's children from physical wreckage. Physical health is still to remain possible in all the future changes of our race. In this work of reconstruction, in accord with the laws of health, professional and individual care of the mouth have no insignificant part to play.

There is a great need of a motive on the part of the people to care for their own teeth and the teeth of their children. There is a deplorable ignorance and inappreciation of the value of good teeth and the harm arising from their neglect. Education is the first and great need, and this may best be accomplished through efforts of dentists, directly and indirectly, with their patients, by publishing papers, in co-operation with philanthropic societies, regular physicians, school boards, and the public schools. These facts especially should be made apparent to all: that the great question of physical welfare, especially so in the case of children, is the question of nutrition; that what is digested and assimilated,

---

[1] E. S. Talbot: Degeneracy: Its Causes, Signs, and Results, Contemporary Science Series.

rather than what one has swallowed, is the principal thing; that the proper mastication of food is an important step in the digestive process; that this is very apt to be thorough, especially with children, in the exact ratio to the condition of the teeth. I asked many children, "On which side do you chew your food?" Immediately came the answers, "On this side, or this; it hurts on the other." Or, "It hurts me to chew with my back teeth. I take a mouthful of food and a swallow of water to help me swallow." How can a child properly masticate its food when one or both sides of his mouth have sensitive, "jumping" shells of teeth, instead of solid bone? Or when his incisors meet in a criss-cross so that his back teeth fall a quarter of an inch or more short of touching? Yet we are told that all degenerations result from a disturbance of nutrition at some critical period of growth. Nathan Oppenheim, in "The Development of the Child," advances strong testimony to his claim that it is nutrition that has far more to do with the mental physical welfare of the child than even heredity. We are told that mortality is greater beyond all comparison from the first to the tenth year of life; that a very large proportion of the physical ills of a lifetime are allotted to the period of childhood; that nearly if not quite one-half of these ills is due to derangement of the digestive apparatus, hence the importance of proper mastication of the food.

Again we are told that the mouth, when rendered foul through the decay of food and teeth, becomes a veritable hotbed for the lodgement and generation of disease germs, an "entrance gate" for infectious diseases. The immunity of the physician, though constantly exposed, is due far more to cleanliness of mouth and person than to anything else. By the lowering of the general tone of the physical condition, the presence of decaying teeth and unclean mouths have much to do, it is asserted by physicians, in the contraction of sickness in general. The dentist and the physician have a grand opportunity for co-operation for the good of public health.

Dr. Edwin Collins, in the *Nineteenth Century* for July, 1899, called attention to the relation of sound teeth to good scholarship. One will readily admit that ability to work well at one's studies necessitates good digestion and freedom from pain. How can a child suffering night after night with toothache do well in school? The answers of the children show that one-fourth of them during the past year suffered a great deal from toothache. "I had the toothache so I could not sleep much nights for two weeks;" "I have had very bad toothache," are some of the statements of the children to me in private. Feeble-minded children, up to seventeen years of age, compare very favorably with normal children in the condition of the teeth, despite the obvious disadvantages of the former in caring for them.[1] The same is more strikingly true in the case of the inferior races. Many children naturally very dull have excellent teeth. We could possibly draw no inference to the effect that naturally good tooth-structure has a direct relation to high mental ability, but who can doubt that the general health and the general condition of the teeth, so far as they mutually affect each other, have an important bearing upon the school work of children?

The children examined were classified by their teachers as bright, average, or dull. Among the children with good teeth there were found thirteen bright children to every ten dull children, but among the children with poor teeth were found only eight bright children to ten dull children. It was found also that very generally the average age of children with good teeth, in any grade, was less than that of children with poor teeth for the same grade. So far as these statistics go, they seem to indicate that the chances of children with good teeth are appreciably better for scholarship and promotion than are those of children with poor teeth.

But, on the other hand, it should be stated here that a very bright child may have very poor teeth, the bad condition of the teeth being, in a considerable measure, induced by over-stimulation of the brain. There seems to be a relation between over-expenditure of nervous energy and imperfect development of the teeth. Every bright, nervous child with not very good teeth should be guarded from over-pressure in school work, especially during the years when the teeth are growing.

A normal increase in height and weight of children is perhaps the best evidence of good physical condition. Many careful measurements of thousands of school children seem to show that, taking a large number of children, there is a relation between superior

---

[1] Dr. Ales Hrdlicka: Report of the Managers of Syracuse State Institutions for Feeble-Minded Children, 1898.

height and weight, and physical, mental, and moral superiority. I refer particularly to the measurements conducted by McDonald in Washington, Klein in Worcester, Porter in St. Louis, Christopher in Chicago, and Byer, of the United States navy. Since nutrition is the basis of development, and since the teeth have an important part to play in the process of digestion, it seemed interesting to know whether any relation could be traced between mental and physical superiority and superiority of the teeth. Owing to the comparatively small number of children who could be grouped for comparison, whatever results were found are not claimed to be perfectly reliable. I do not wish to be understood as making any broad generalization on the data gathered, but the results certainly deserve serious consideration.

The weight of boys with good teeth was compared with the weight of boys with poor teeth at each age from five to fourteen, also the weight of girls with good teeth with the weight of girls with poor teeth of corresponding-ages, there being twenty groups in all. The children with good teeth surpassed children with poor teeth in weight in fifteen out of the twenty groups. Combining the average weights of boys and girls with good teeth for each age, and the weights of boys and girls with poor teeth for corresponding ages, and comparing, it was found that the children with good teeth surpassed in weight the children with poor teeth at all ages except at eight and thirteen. Combining the averages of children with good teeth for all ages and the averages of children with poor teeth for all ages, the children with good teeth surpassed the children with poor teeth in weight by an average of 2.7 pounds per child. In other words, children with good teeth were, on the average, about half a year ahead of the children with poor teeth in physical development, as shown by weight.

Parents should know, also, that the calcification of the teeth begins long before the child is born; that any disturbance of nutrition due to bad heredity or maternal impressions becomes registered upon the teeth; that the enamel-organs and dentine germs of the permanent teeth form before birth; that the first permanent molars begin to calcify before birth; that from birth to five years of age is the critical period for the calcification of the permanent teeth. It is the general notion that it is of little moment whether the baby teeth decay or not. So far as they affect the general health and condition of the mouth, the baby teeth have a very great influence upon the permanent teeth. So far as all the permanent teeth except the wisdom-teeth are concerned, their natural fate is fixed before the child has cut his permanent front teeth.

The following diagram [1] of the teeth is instructive. The lines show what teeth and what portion of each tooth may suffer from malnutrition at certain ages. To find what teeth and what portions may suffer at three years of age, for example, follow along the three-year line. It will be seen that the central and lateral incisors will suffer just below the middle of the enamelled part, the cuspid at the end, and the sixth-year molar.

In spite of the fact that the early years and the care of the temporary teeth are so important, it was very rare to find that the temporary teeth had been particularly cared for. Of the eighteen hundred and forty cavities in the baby teeth, only forty-eight had been filled,—*i.e.,* 2.6 in each hundred. Very few of the children under six years of age brushed their teeth or had them brushed. There is no reason why children of three or four years of age may not brush their own teeth, even so acquire the habit that they will feel uncomfortable when their teeth are not clean.

Dr. W. D. Miller, of Berlin, has shown that there is a microorganism concerned in the decay of the teeth. These microbes, infectious in character, lodge upon some hollow or unclean portion of the tooth. They secrete an acid which dissolves the enamel of the tooth, and they burrow gradually deeper and deeper into the tooth. It is supposed that these microbes do not live upon the tooth-substance, but upon the sugar and starchy foods which have been acted upon by the saliva of the mouth, while the acid secreted caused the crumbling away of the tooth. Hence the importance of keeping the teeth perfectly clean. A tooth that is smooth and perfectly clean can scarcely decay.

[1] Reproduced from Degeneracy: Its Causes, Signs, and Results, E. S. Talbot, Contemporary Science Series.

Parents should know of the effect of different foods upon the teeth. Unlike other portions of the body, after the enamel has once been formed there is no cell for nourishing or repairing the enamel. Whatever effect the food is to have upon the formation of enamel of the teeth must take place before a certain age. In the case of infants, next to mother's milk, cow's milk diluted with barley-water is the best food for the teeth. When the child is old enough, the teeth may be well nourished by means of rolled oats, rolled wheat, graham, meat, fish, eggs, and fruits. The natural order of food for man, according to Dr. Röse, is the following: Flesh, whole grains, vegetables, bread, cooked meat, butter, fat. Races which live largely upon flesh are least afflicted with caries.

Children should not be fed largely upon soft and sloppy foods. The strong exercise of the teeth in mastication seems to be the natural and necessary accompaniment of sound teeth. Children should have something to chew upon. It is significant that an examination by Dr. W. Smith of the Sioux Indians in Buffalo Bill's Wild West Show disclosed the fact that they were entirely free from caries of the molars and premolars, but that the molars were worn.[1] The same was found to be true in the case of the teeth of the Egyptian skulls brought to England by Dr. Petrie. Dr. Smith claims that the grinding surfaces of sound molars are worn, but diseased molars are not worn, and that this fact is strong testimony to the necessity of vigorous exercise of the teeth in maintaining a healthy condition.

Even the degree of the hardness of the water is of importance. I am aware that the influence of the drinking-water upon the teeth is considered of no importance by some authorities, but it is a fact worth notice that in the extensive investigation in Germany there appeared a wide margin in favor of the teeth of children in regions rich in lime: two thousand nine hundred and seventy-three children examined in a region poor in lime showed that 34.9 per cent. of all teeth were diseased; an examination of two thousand seven hundred and eight children in a region rich in lime showed that only 16.7 per cent. of the teeth were diseased. It is interesting to note that the degree of the hardness of the water in the region most afflicted with caries was 0.5; in the region of better teeth, 8 to 12. The degree of the hardness of water in Andover is

[1] Journal of Anthropological Institute, vol. xlvi., p. 446.

from 1.3 to 1.7. The percentage of diseased teeth among Andover school children is 31.4, which corresponds very nearly to the result of the German investigation for districts of equally soft water.

The part that the teeth have to play in personal appearance is by no means unimportant. A beautiful set of teeth adds very much to a face otherwise attractive, but no face can be said to be attractive which is disfigured by a poorly kept and irregular set of teeth. Malformation of the mouth and teeth may cause undue self-consciousness and diffidence on the part of the possessor, even a moroseness of disposition, which greatly hinders his happiness and success in life. By a sort of intuition which we could scarcely rid ourselves of if we would, we are compelled to discount people with deformities. It is a great pity that parents should allow a child to bear the extra burden, in his search for success and happiness, of deformity in any degree that is within their power to have remedied. A careful dentist tells me that about ninety per cent. of the various deformities found in the Andover children may be remedied wholly or in a great measure if undertaken in due season.

That the decay of the teeth may cause deafness is well understood by aural surgeons. Dr. Samuel Sexton, before quoted, says, "I have long been in the habit of examining the teeth of children brought to me with aural diseases, and it happens very often that unsuspected dental irritation is found to coexist to which the aural irritation is in some measure attributable. Among the large number of school children who attend the aural clinics at the infirmary it is rare to find one where dental irritation should not be considered as a causative factor."

It seems to me that the dentist has a responsibility in this condition of the teeth of school children. His part is more than to receive children in his office, do his work honestly and well, and collect his fees. With the dentist rests naturally the burden of the general enlightenment of the public. Whose fault is it that the great majority of parents, even those fairly well educated, never dream that their children have any permanent teeth until they see the incisors coming, much less that their child of eight has had four permanent molars for two years, and that two or even three of the four are already diseased? "Mamma knows about that tooth," said a little girl to me, when I called attention to a cavity in one of her first permanent molars, "but she is not going to have it filled, because it is a baby tooth." The record of these first molars shows that this mother, apparently more thoughtful than most of them, because she did intend to have the permanent teeth filled, was only one of a great majority who believe that the sixth-year molar is a baby tooth.

No one dentist may perhaps take upon himself, for obvious reasons, the enlightenment of the public in regard to the care of the teeth, especially so far as it concerns his profession, but an association of dentists, in any community, may and ought to do so. There are many ways by which this might be done, it seems to me, in a town like Andover: timely articles in the local paper on the teeth and their care, by the members of the association; co-operation with the regular physicians. Mothers' meetings are common now even in small towns,—a timely talk to mothers on the care of the teeth of infants and young children would be gratefully received, I am sure. The association should know what instruction is given in the public schools in regard to the teeth, what text-books are used, what charts, etc. Superintendents, I believe, would welcome any suggestions from dentists in this matter. Every town has its characteristic local conditions. Perhaps a manual for the use of the schools could be prepared, which would be far superior to the treatment in any text-book that could be purchased for that town. In some of the more progressive towns, dental inspection of school children might be established through the efforts of the local dentists. This must come in time. The day is approaching when every child in our public schools will be considered as a being with a body as well as with a mind. With the burden of public enlightenment comes to the dentist also the responsibility of the policy or lack of policy of public officials in this matter, so far as it concerns public institutions, especially the public schools. If this responsibility rests not on the dentist, then on whom? And here comes up the question of the poor. Dr. Richard C. Newton, in the *Dental Cosmos* for May, 1896, says, "The dentists, if they wish to be esteemed by the public generally as specialists of medicine, must give of their time and skill to treating the poor. It is the willingness to give thought, time, and skill to the service of the poor which has elevated and ennobled the profession of medicine. It is this that has made it the most generally beloved and respected of all the professions." How widely the free clinics for the poor have been established and how greatly abused I do not know, but there are still many deserving children without them. As one result of this work in the Andover schools, there will be established, mainly through the generosity and philanthropic spirit of our local dentists, a dental room, open at least three forenoons a month, to the service of the children of the poor.

I desire, in closing, to acknowledge my great indebtedness to the dentists of Andover, Drs. Gilbert, Hulme, and MacIntosh, for their very generous assistance and helpful suggestions. Without many hours of hard work on their part so freely given, this investigation could hardly have been conducted. I am indebted, also, to Dr. William H. Burnham and Librarian Louis N. Wilson, of Clark University, for suggestions and aid in securing literature on the subject.

## Figures and Tables

**Figure f1:**